# Gut microbiota in mucosa and feces of newly diagnosed, treatment-naïve adult inflammatory bowel disease and irritable bowel syndrome patients

**DOI:** 10.1080/19490976.2022.2083419

**Published:** 2022-06-13

**Authors:** Hana Čipčić Paljetak, Anja Barešić, Marina Panek, Mihaela Perić, Mario Matijašić, Ivana Lojkić, Ana Barišić, Darija Vranešić Bender, Dina Ljubas Kelečić, Marko Brinar, Mirjana Kalauz, Marija Miličević, Dora Grgić, Nikša Turk, Irena Karas, Silvija Čuković-Čavka, Željko Krznarić, Donatella Verbanac

**Affiliations:** a Center for Translational and Clinical Research, University of Zagreb School of Medicine, Zagreb, Croatia; b Division of Electronics, Ruđer Bošković Institute, Zagreb, Croatia; c Department for Virology, Croatian Veterinary Institute, Zagreb, Croatia; d Department of Internal Medicine, Unit of Clinical Nutrition, University Hospital Centre Zagreb, Zagreb, Croatia; e University of Zagreb School of Medicine, Zagreb, Croatia; f Department of Internal Medicine, Division of Gastroenterology and Hepatology, University Hospital Centre Zagreb, Zagreb, Croatia; g Department of Gastroenterology, Hepatology and Clinical Nutrition, University Hospital Dubrava, Zagreb, Croatia

**Keywords:** Gut microbiota, gut mucosa, fecal microbiota, inflammatory bowel disease, Crohn’s disease, ulcerative colitis, irritable bowel syndrome, treatment-naïve patients

## Abstract

The knowledge on how gut microbes contribute to the inflammatory bowel disease (IBD) at the onset of disease is still scarce. We compared gut microbiota in newly diagnosed, treatment-naïve adult IBD (Crohn’s disease (CD) and ulcerative colitis (UC)) to irritable bowel syndrome (IBS) patients and healthy group. Mucosal and fecal microbiota of 49 patients (13 UC, 10 CD, and 26 IBS) before treatment initiation, and fecal microbiota of 12 healthy subjects was characterized by 16S rRNA gene sequencing. Mucosa was sampled at six positions, from terminal ileum to rectum. We demonstrate that mucosal microbiota is spatially homogeneous, cannot be differentiated based on the local inflammation status and yet provides bacterial footprints superior to fecal in discriminating disease phenotypes. IBD groups showed decreased bacterial diversity in mucosa at all taxonomic levels compared to IBS. In CD and UC, *Dialister* was significantly increased, and expansion of *Haemophilus* and *Propionibacterium* characterized UC. Compared to healthy individuals, fecal microbiota of IBD and IBS patients had increased abundance of Proteobacteria, *Enterobacteriaceae*, in particular. Shift toward reduction of *Adlercreutzia* and butyrate-producing taxa was found in feces of IBD patients. Microbiota alterations detected in newly diagnosed treatment-naïve adult patients indicate that the microbiota changes are set and detectable at the disease onset and likely have a discerning role in IBD pathophysiology. Our results justify further investigation of the taxa discriminating between disease groups, such as *H. parainfluenzae, R. gnavus, Turicibacteriaceae, Dialister*, and *Adlercreutzia* as potential biomarkers of the disease.

## Introduction

Crohn’s disease (CD) and ulcerative colitis (UC) represent two clinically and morphologically distinct entities of inflammatory bowel disease (IBD) that are, together with irritable bowel syndrome (IBS), the most common, life-long gastrointestinal disorders with serious impact on the patient’s quality of life. Due to their high prevalence, IBD and IBS impose a significant economic burden, in North America and Europe, in particular.^1–4^

IBD and IBS share similar symptomatology and demographics, so detailed clinical examination including assessment of laboratory, histological, endoscopic, and radiological features is needed for their differentiation. While IBD is characterized by mucosal inflammation, there are no clear causative anatomical or biochemical deviations that can be used for diagnosis of IBS.^5^ Multifactorial etiology of IBD is characterized by the sustained immune response toward altered or pathogenic microbiota within a genetically susceptible host.^6,7^ It is likely that, rather than presence of particular pathogens, alterations in composition, and diversity of gut microbiota (dysbiosis) play an important role in the onset and progression of the disease.^8,9^ As for IBD, the complex etiology of IBS involves genetic, immune, environmental, neurological, and psychological factors.^5^

The intricate symbiotic relationship of the host and resident gut microbiota community provides the host with multiple essential functions and plays a crucial role in the maintenance of health. High inter-individual differences, as well as quantitative variation in the gut microbiota composition under the influence of a large number of host and environmental factors (including food intake, medication, geographical location, age, etc.),^10–12^ present a challenge in defining what constitutes a “normal” human microbiome. The most dominant bacterial phyla found in a healthy human gut are Bacteroidetes, Firmicutes, and Actinobacteria.^13,14^ This complex landscape can be stratified into reproducible patterns of variation of major taxa (i.e. *Bacteroides, Prevotella*, and *Ruminococcus*) in fecal metagenomes termed enterotypes.^15^ Recognizing compositional patterns and separating the human population across these three possible configurations can help in understanding human health and disease conditions.^16^

Major shifts in the gut microbial composition have been reported in IBD patients, with reduced diversity and decrease in members of Firmicutes phylum (largely due to reduction in abundance of *Clostridia* clusters IV and XIVa, as well as *Eubacterium*), and simultaneous expansion in Proteobacteria (*Enterobacteriaceae*, in particular).^8,17–19^ In addition, dysbiosis is found in other microbial constituents (e.g. fungi, viruses, archaea).^9,20^ Changes in microbial composition lead to alterations in microbiome metabolic pathways and levels of gut microbiota derived metabolites, such as bile acids, short-chain fatty acids (SCFAs), and tryptophan metabolites, which have been implicated in the pathogenesis of IBD.^21,22^

Gut microbes are important contributors to the IBD onset, yet studies investigating mucosa-associated bacteria at the start of the disease are rare. Majority of reports focused on the analyses of fecal microbiota composition, but a number of recent studies investigated gut mucosa-associated microbial communities, either independently^23,24^ or comparing tissue and fecal microbiota of the same individuals.^25–31^ Several studies investigated both feces and mucosa of the new-onset CD and UC in pediatric patients.^27,32,33^ As comprehensive studies on treatment-naïve, newly diagnosed adult patients with IBD are scarce, present report represents our attempt to fill this gap.

The recruited IBD population (both CD and UC) were undiagnosed at the time of study inclusion and not treated with antibiotics or anti-inflammatory therapy. The chosen study design enables insight into the gut microbiota status prior to any influences of treatment protocols. Although our study cohort is of limited size, it provides the most detailed coverage of the mucosal bacterial content along the gut length in CD, UC and IBS patients, parallel analysis of fecal bacteria in the same individuals, as well as a comparison to fecal microbiota community of healthy individuals.

## Results

At the time of inclusion in the study, the patients reported gut discomfort with no previous diagnosis of a gastrointestinal disease and did not receive anti-inflammatory or antibiotic treatment. Initially, 93 participants were recruited: 81 undergoing IBD and IBS diagnostic procedures and 12 healthy volunteers to serve as a control group. After excluding participants with other or missing diagnoses, participants with missing/low quality samples as well as after IBS-IBD age and sex matching, the final study cohort consisted of three groups with 61 participants: Crohn’s disease (CD group, n = 10), ulcerative colitis (UC group, n = 13), and IBS control group (IBS group, n = 26). Healthy controls (H group, n = 12) were included for feces sampling only (Supplementary Figure S1). According to endoscopic scores, the majority of patients had mild to moderate IBD activity, with one CD patient presenting severe disease. CD group had a higher median age and higher representation of female participants (Table 1).

**Table 1.** Demographic data, clinical indices, biochemical and fecal markers, and phenotypes according to the Montreal classification

	CD	UC	IBS	Healthy
N	10	13	26	12
Demographic data				
Median age at diagnosis (range)	46 (21–72)	31 (18–54)	31 (19–56)	35 (24–56)
Female, n (%)	7 (70)	6 (46)	15 (58)	6 (50)
BMI median (range)	24.0 (19.7–31.6)	23.0 (18.4–32.0)	23.2 (18.5–33.2)	24.6 (22.9–28.9)
Biochemical parameters				
CRP, mg/L median (range)	2.2 (0.5–8.3)	1.0 (0.4–17.5)	0.8 (<0.3–18.5)	
Fecal calprotectin mg/kg median (range)	114 (<20-348)	516 (21–1800+)	29 (<20-373)^#^	
Disease activity*				
Mild	4	5		
Moderate/severe	4/1	7		
Unknown	1	1		
Montreal classification				
Age at diagnosis				
A1 < 17 years	0	0		
A2 17–40 years	3	10		
A3 > 40 years	7	3		
Location/extension				
L1 ileal^a^/E1 proctitis^b^	2	7		
L2 colonic^a^/E2 left-sided colitis^b^	4	2		
L3 ileocolonic^a^/E3 extensive colitis^b^	3	3		
Unknown^†^	1	1		
Behavior CD				
B1 non-stricturing, non-penetrating	5			
B2 stricturing	2			
B3 penetrating	0			
B2p perianal modifier	1			
B3p perianal modifier	1			
Unknown^†^	1			

*Endoscopic disease activity at the diagnosis was assessed according to SES endoscopic score for CD and Mayo endoscopic score for UC.^34^

^#^Missing data in 50% of patients.

^†^Location and behavior were determined during endoscopy, and two patients had no inflamed sites but were subsequently diagnosed as CD and UC.

^a^CD

^b^UC

Microbiota composition was determined in gut mucosa specimens of 49 patients (CD, UC, and IBS): 46 sampled at six positions from terminal ileum to rectum (three patients had an additional sample taken to compare an inflamed-uninflamed pair at the same position), with five positions sampled in remaining three patients, resulting in 294 mucosa samples. Sampling scheme for IBD patients only with respective inflammation status is shown in Supplementary Figure S2. Two UC patients had a discontinued inflammation, with “caecal patch” lesions with aphthous ulcerations in the cecum. In one UC and one CD patient, all six positions were endoscopically observed as uninflamed. However, inflammation was subsequently detected in histological analysis of both patients, and the CD patient had MR enterography features of active Crohn’s disease. All six positions were sampled from 26 IBS individuals, with no inflamed sites along the gut.

### Gut mucosa microbiota profiles from terminal ileum to rectum

**Figure 1.**
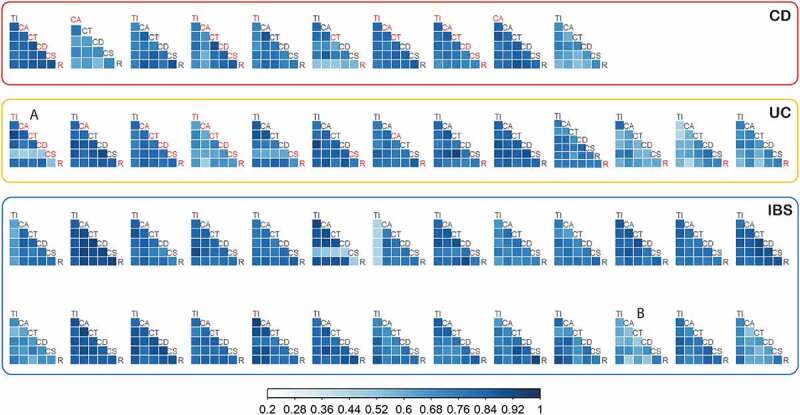
Spearman correlation of bacterial abundances (family level) among positions along the gut within each patient: terminal ileum (TI), ascending colon (CA), transverse colon (CT), descending colon (CD), sigmoid colon (CS), and rectum (R). Inflamed positions are labeled in red. For patients A and B, relative abundance profiles are presented in Supplementary Figure S3. CD – Crohn’s disease, UC – ulcerative colitis, IBS – irritable bowel syndrome.

**Figure 2.**
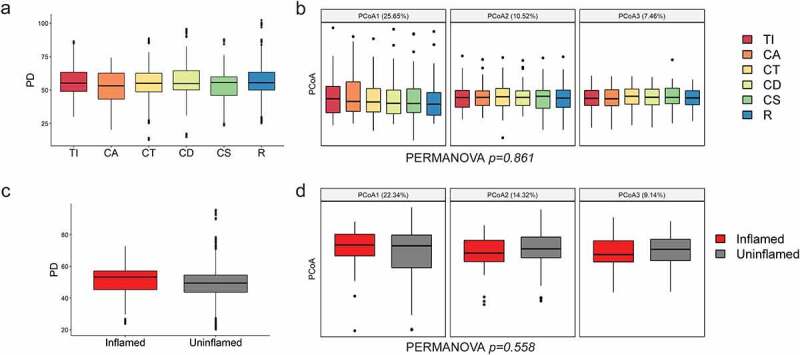
Microbial diversity of intestinal mucosa at six locations along the gut, colored by position (a, b) and inflammation status (c, d). Terminal ileum (TI), ascending colon (CA), transverse colon (CT), descending colon (CD), sigmoid colon (CS), and rectum (R). (a, c) Alpha diversity measured by phylogenetic diversity PD. B.-H. corrected Wilcoxon test between categories showed no significance. (b, d) The first three principal coordinates of weighted UniFrac (percentage of the variation explained in brackets) are shown for beta diversity. Bonferroni-corrected t-test between categories showed no significance.

Mucosal microbiota composition along the colon of each individual did not vary significantly at the family level (Figure 1). Bacterial abundances correlated well (median Spearman’s rho per patient was between 0.602 and 0.905) among positions from terminal ileum to rectum within each patient. There were a few subjects with lower correlations of microbiota profiles between positions (the lowest rho of 0.47), but these cases demonstrated relative abundance profiles in good concordance among samples, as exemplified for one UC and one IBS patient (marked A and B in Figure 1, respectively) shown in detail in Supplementary Figure S3. No significant trends were observed among sampling positions along the colon in terms of alpha and beta diversity (Figure 2a-b, Supplementary Figure S4), further confirmed by homogeneity of variances (betadisper test, p-value = 0.62). To further test the consistency of mucosal microbiota profiles, we compared the abundances of bacterial families at two most distant anatomical sites (i.e. terminal ileum and rectum) and expectedly found no significant variation in abundance among families (Supplementary Table S2).

### Effect of inflammation on microbiota composition in IBD

Despite inflammation of the gut mucosa defined as one of the main diagnostic criteria in IBD, we observed high agreement in alpha and beta diversity of inflamed and uninflamed sites (Figure 2c-d, for other alpha indices, see Supplementary Figure S5), with no significant heterogeneity in variances (betadisper test, p-value = 0.40). In addition, we have pairs of inflamed and uninflamed samples taken at the same gut position from three participants: at CA in one CD and one UC patient and at R position in another UC patient (Supplementary Figure S2.b). These inflamed-uninflamed pairs displayed a marked concordance of bacterial profiles on the family level, with high correlation coefficients of 0.84, 0.82, and 0.76, respectively. Furthermore, we have not detected significant inflammation-dependent abundance changes of any bacterial taxa (Supplementary Table S3).

### Microbial diversity in gut mucosa of IBD patients

**Figure 3.**
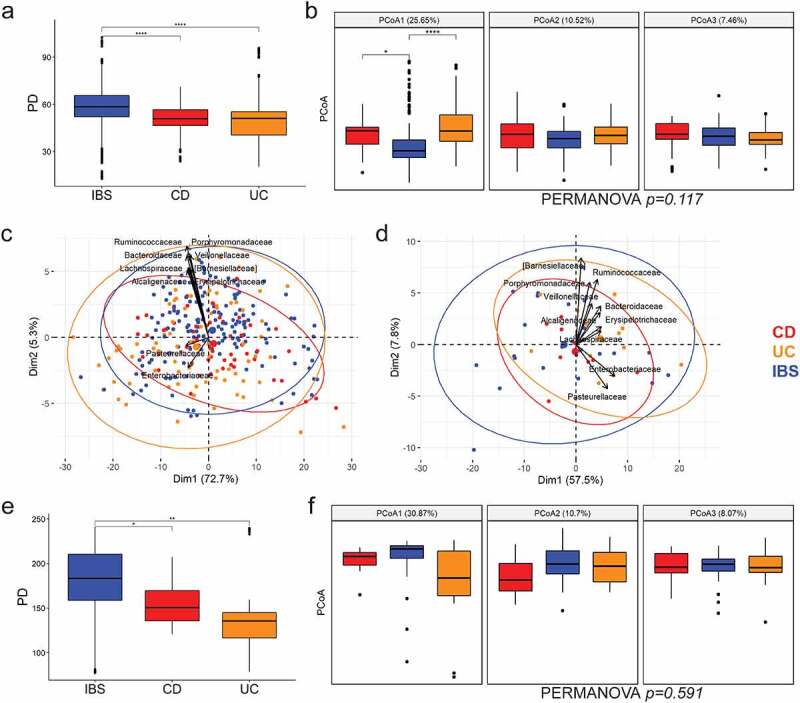
Microbial diversity of intestinal mucosa at six positions along the gut (a-c), and after merging sequences for all positions in the patient-specific profiles (d-f). Alpha diversity measured by phylogenetic diversity PD, with B.-H. corrected Wilcoxon test between categories. (a, e). The first three principal coordinates of weighted UniFrac (percentage of the variation explained in brackets) are shown for beta diversity (b, f), with Bonferroni-corrected t-test between categories. First two principal components in PCA and loadings for top ten families are shown before (c) and after position merging (d). CD – Crohn’s disease, UC – ulcerative colitis, IBS – irritable bowel syndrome. *p < .05, **p < .01, ***p < .001, ****p < .0001.

Microbiota diversity decreased in mucosa of CD and UC patients compared to the IBS, on the level of both alpha (Figure 3, panels a and e, Supplementary Figure S6) as well as beta diversity. PCoA1, encompassing 25% of variance, corresponded well to IBS versus IBD samples (particularly IBS versus UC) (Figure 3b), followed by a significant variance heterogeneity was observed between the three diagnoses (betadisper test p-value = 0.03).

Considering that the sampling position along the gut and the inflammation status of the sample played a small role in microbiota composition (Figures 2 and 3), displaying high concordance within each patient and revealing no taxa with significant differences in abundance, individual raw sequences of each patient were pooled in a single sample for downstream analyses. In addition, the low sequencing depth obtained for a fraction of samples affected observed microbial diversity (Supplementary Figure S7), but the distribution of detected families was consistent with samples of the same person that had higher sequencing depth. Therefore, rather than eliminating the samples with suboptimal depth, pooling allowed mitigating this source of variation, in turn creating a unique and gut-wide patient-specific profile of mucosa microbiota and subsequent comparison to their fecal profiles.

The sample pooling did not affect the observed diversity trends among patient cohorts, with IBD still displaying decreased alpha diversity compared to IBS (Figure 3e). Similarly, the driver of the variance was the difference of microbiota profiles between IBD and IBS, especially UC and IBS (Figure 3f). Crucially, the same families (displayed with arrows) drove the disease-specific variance on both per position and pooled data, as demonstrated by the PCA biplot loadings (Figure 3c-d).

The abundance of individual taxa, presented in Table 2, revealed families and genera driving the difference between patient groups based on the effect size (>0.400). Compared to IBS, *Dialister, Propionibacterium* and *Haemophilus* were significantly enriched in IBD (p < .05), owing to an increase in UC patients. UC samples had a marked increase in *Enterobacteriaceae, Neisseriaceae, S24-7*, and decrease in *Coriobacteriaceae* (particularly in comparison to CD). Higher abundance of *Lachnospiraceae* and *Erysipelotrichaceae* was a marker of CD, reaching statistical significance when compared to the UC group. Compared to IBS, CD samples were significantly depleted in *Alcaligenaceae, Desulfovibrionaceae, Phascolarctobacterium*, and *Oscillospira*, with indicative reduction in *Verrucomicrobiaceae* and *Adlercreutzia*. *Oscillospira*, with indicative reduction in *Verrucomicrobiaceae* and *Adlercreutzia*.

**Table 2.** Taxa abundance trends in mucosa profiles (at family and genus levels) between patient groups presented as effect size (ES) and uncorrected p-values in Kruskal-Wallis test. ** p < .01; * p < .05; I p < .1. Negative ES values indicate higher abundance in the first group of pairwise comparison. ES larger than 0.4 are presented in boldface

			IBD vs IBS		CD vs UC		CD vs IBS		UC vs IBS	
Phylum	Family	Genus	ES	P	ES	P	ES	P	ES	P
Firmicutes	*(Mogibacteriaceae)*		−0.025		0.291		0.458	*	−0.238	
	*Lachnospiraceae*		−0.160		**−0.724**	*	0.122		0.186	
		*(Ruminococcus)*	−0.032		**−0.571**	I	−0.187		0.270	
		*Blautia*	−0.124		**−0.557**	I	−0.026		0.207	
		*Coprococcus*	−0.088		**−0.465**		−0.019		0.238	
		*Dorea*	−0.084		**−0.511**		0.030		0.248	
		*Roseburia*	0.062		−0.315		0.176		0.342	
	*Ruminococcaceae*		−0.140		−0.144		0.262		−0.011	
		*Oscillospira*	0.049		−0.114		0.330	*	0.185	
	*Veillonellaceae*		−0.095		−0.165		0.375	I	−0.017	
		*Dialister*	**−0.573**	**	−0.265		**−0.497**	I	**−0.496**	*
		*Phascolarctobacterium*	0.366	I	0.137		**0.543**	*	0.337	
	*Erysipelotrichaceae*		−0.105		**−0.865**	*	−0.061		0.235	
Bacteroidetes	*(Barnesiellaceae)*		−0.340	I	−0.018		−0.108		−0.354	
	*Bacteroidaceae*		0.013		**−0.476**	I	**0.413**		0.235	
		*Bacteroides*	0.033		**−0.485**	I	0.260		0.252	
	*Porphyromonadaceae*		0.001		−0.117		0.286		0.206	
	*Rikenellaceae*		0.140		0.085		**0.438**	I	0.130	
	*S24-7*		−0.227		**0.521**	I	0.273		**−0.536**	*
Proteobacteria	*Alcaligenaceae*		0.027		0.147		**0.432**	*	0.021	
		*Sutterella*	0.025		0.138		**0.401**	I	0.103	
	*Neisseriaceae*		−0.338	I	0.249		−0.099		−0.369	I
	*Desulfovibrionaceae*		0.097		0.101		0.428	*	0.087	
		*Bilophila*	0.013		0.109		0.315	I	0.019	
		*Desulfovibrio*	0.019		−0.209		0.042		0.096	
	*Enterobacteriaceae*		−0.345	I	0.267		−0.046		−0.462	I
	*Pasteurellaceae*		**−0.452**	*	0.311		−0.081		**−0.645**	*
		*Haemophilus*	**−0.428**	*	0.292		−0.114		**−0.574**	*
	*Xanthomonadaceae*		**−0.421**	I	−0.049		−0.200		**−0.408**	
Actinobacteria	*Nocardioidaceae*		−0.392	I	−0.236		−0.334		−0.374	
		*Aeromicrobium*	**−0.418**	I	−0.215		−0.346		−0.372	
	*Propionibacteriaceae*		**−0.449**	*	0.016		−0.181		**−0.531**	*
		*Propionibacterium*	**−0.451**	*	0.022		−0.361		**−0.471**	*
	*Coriobacteriaceae*		−0.045		**−0.489**		0.186		0.173	
		*Adlercreutzia*	0.075		0.396		**0.469**	I	−0.101	
		*Eggerthella*	−0.181		**−0.487**		−0.190		0.055	
Verrucomicrobia	*Verrucomicrobiaceae*		0.137		0.252		**0.514**	I	−0.039	
Spirochetes	*Leptospiraceae*		−0.327		−0.241		−0.282		−0.333	
		*Leptospira*	−0.360	I	−0.227		−0.279		−0.311	

**Figure 4.**
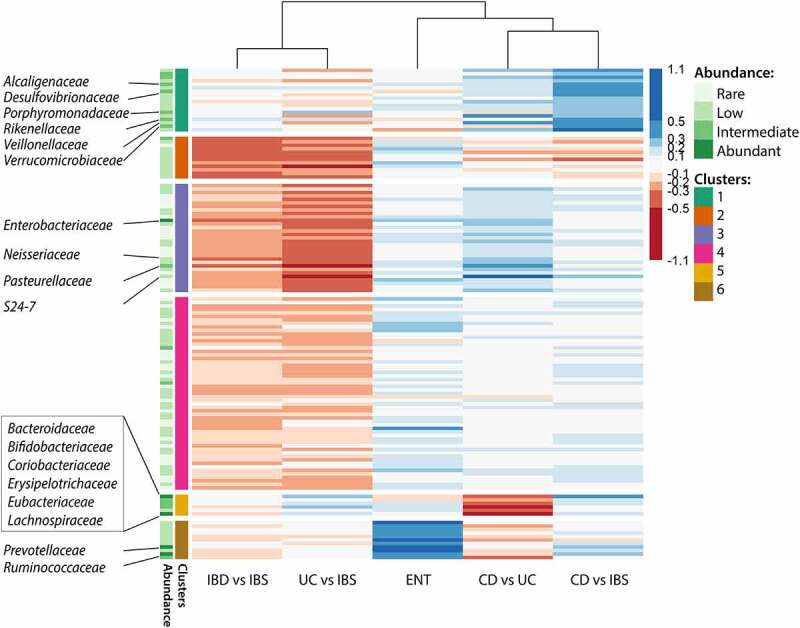
Heatmap of family level effect sizes in pairwise comparisons of mucosal profiles. K-means clustering of families with six clusters. Dendrogram on top shows clustering based on distance of effect size profiles, for each pairwise comparison. CD – Crohn’s disease, UC – ulcerative colitis, IBS – irritable bowel syndrome, ENT – *Bacteroides* versus *Prevotella* dominated enterotype.

Clustering of these effect sizes revealed grouping of UC-based and CD-based changes (Figure 4). The families abundantly found in gut mucosa were mostly grouped in cluster 1 and were predominantly reduced in CD, and cluster 5 differentiating CD from UC. Furthermore, clusters 3 and 4 significantly differentiated between IBS and UC patients, with cluster 3 comprising families markedly enriched in UC. Cluster 1, associated with differences between CD and IBS patients predominantly comprised families depleted in CD, while cluster 5 differentiated CD from UC with six families less abundant in mucosa of UC patients. Enterotype-associated cluster 6 differentiated *Bacteroides* from *Prevotella*-dominated microbiota community.

### Microbiota diversity and composition in feces

**Figure 5.**
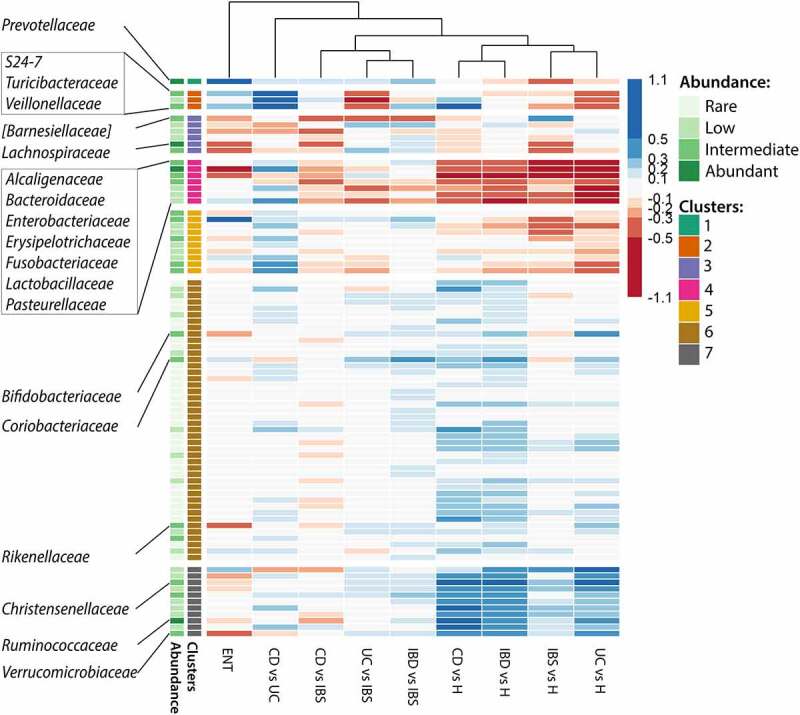
Heatmap of clustered family level effect sizes in pairwise comparisons of fecal profiles, based on k-means clustering of families with seven clusters. Dendrogram on top shows clustering based on distance of effect size profiles, for each pairwise comparison. CD – Crohn’s disease, UC – ulcerative colitis, IBS – irritable bowel syndrome, H – healthy, ENT – *Bacteroides* versus *Prevotella* dominated enterotype.

Differential abundances in fecal microbiota of the same patient cohort and the additional group of healthy donors are shown in Table 3. The heatmap in Figure 5 revealed a shared pattern of healthy fecal profiles (four columns on the right), followed by the trends in the IBS-driven set of comparisons (columns 3–5). Several clusters differentiated patients from healthy donors: clusters 4 and 5 with families more abundant in disease groups, and cluster 7 with families depleted in both IBS and IBD. At the same time, cluster 2 displays UC-associated taxonomic divergence. Of note, *Prevotellaceae* as a single family in cluster one are the driver of enterotype-associated footprint.

**Table 3.** Taxa abundance trends at family and genus levels in fecal profiles between patient groups and healthy controls presented as effect size (ES) and uncorrected p-values in Kruskal-Wallis test; ** p < .01; * p < .05; I p < .1. Negative ES values indicate higher abundance in the first group of pairwise comparison. ES larger than 0.4 are presented in boldface

			IBD vs H		CDvsH		UCvsH		IBSvsH		IBDvsIBS		CDvsUC		CDvsIBS		UCvsIBS	
Phylum	Family	Genus	ES	p	ES	p	ES	p	ES	p	ES	p	ES	p	ES	p	ES	p
Bacteroidetes	*Bacteroidaceae*		−0.315		−0.324		**−0.612**	I	**−0.613**	*	0.039		**0.448**		−0.255		−0.134	
		*Bacteroides*	**−0.419**		−0.353		**−0.590**	I	**−0.602**	*	−0.057		0.287		**−0.417**		−0.159	
	*Prevotellaceae*		−0.112		−0.077		−0.195		−0.339	*	0.223		0.191		0.164		0.120	
		*Prevotella*	−0.130		−0.071		−0.177		−0.374	*	0.196		0.179		0.147		0.104	
	*Porphyromonadaceae*		−0.022		−0.179		−0.111		**−0.451**	I	0.158		−0.123		−0.192		0.243	
		*Parabacteroides*	−0.126		−0.181		−0.069		**−0.418**	I	0.128		−0.173		−0.333		0.219	
	*(Paraprevotellaceae)*		−0.125		−0.044		−0.198		**−0.400**	I	0.222		0.146		0.159		0.144	
		*(Prevotella)*	−0.209		−0.179		−0.227		**−0.633**	**	0.201		0.078		0.095		0.181	
	*(Barnesiellaceae)*		−0.010		−0.118		−0.018		0.309		−0.332		−0.073		**−0.464**	*	−0.312	
Firmicutes	*Lachnospiraceae*		0.040		−0.164		−0.005		**−0.406**		0.158		−0.055		−0.392	I	0.015	
		*Blautia*	0.159		0.183		0.209		−0.298		0.219		−0.157		**−0.481**	I	0.078	
		*Coprococcus*	0.224		0.080		0.362		−0.197		0.147		−0.380		**−0.462**	I	0.132	
		*(Ruminococcus)^a^*	−0.375		**−0.608**	I	−0.238		−0.374		−0.126		−0.348		**−0.771**	**	−0.082	
		*Roseburia*	0.290		0.320		0.207		−0.233		0.396	I	0.057		0.132		0.268	
		*Anaerostipes*	**0.759**	**	**0.973**	**	**0.611**	*	0.256		0.236		0.180		0.218		0.155	
	*Ruminococcaceae*		**0.489**	I	**0.504**		0.349		0.117		0.135		0.087		−0.271		0.055	
		*Ruminococcus*	**0.466**	*	**0.408**		**0.540**	*	0.203		0.038		−0.244		**−0.432**	I	0.095	
		*Fecalibacterium^b^*	0.243		0.293		0.316		0.038		−0.047		0.048		−0.120		−0.090	
	*Veillonellaceae*		0.091		**0.550**	I	**−0.414**		−0.213		0.239		**0.914**	**	0.079		−0.312	
		*Phascolarctobacterium*	**0.568**		**0.738**	*	0.288		0.057		−0.274		−0.066		**−0.559**	*	−0.299	
		*Dialister*	**−0.571**	I	**−0.659**	I	**−0.494**		−0.158		0.253		0.051		0.208		0.094	
		*Veillonella*	−0.310		−0.235		**−0.406**		−0.386	I	0.079		0.200		0.169		−0.134	
	*Christensenellaceae*		**0.559**	*	**0.581**	I	**0.545**	I	0.260		0.171		0.083		0.091		0.104	
	*(Mogibacteriaceae)*		**0.417**	I	**0.425**		0.345		0.086		0.188		0.114		0.029		−0.114	
	*Dehalobacteriaceae*		**0.448**	I	**0.417**		**0.415**		0.225		0.185		0.081		−0.008		0.147	
	*Erysipelotrichaceae*		−0.368		−0.377		−0.373		−0.216		−0.151		−0.193		**−0.471**	*	−0.135	
		*(Eubacterium)*	**−0.568**	*	**−0.580**	I	**−0.532**	I	**−0.583**	*	0.023		0.200		−0.146		0.092	
	*Turicibacteraceae*		−0.085		0.243		**−0.483**	I	−0.024		−0.111		**0.708**	*	0.107		**−0.529**	*
		*Turicibacter*	−0.149		0.293		**−0.482**	I	−0.028		−0.135		**0.604**	*	0.076		**−0.567**	*
	*Lactobacillaceae*		**−0.480**	I	**−0.448**		**−0.563**	*	**−0.421**	I	−0.088		0.071		−0.123		−0.134	
		*Lactobacillus*	**−0.511**	*	**−0.432**		**−0.593**	*	**−0.515**	*	−0.082		0.129		−0.164		−0.101	
Proteobacteria	*Alcaligenaceae*		−0.399	I	−0.344		**−0.742**	*	**−0.822**	**	0.100		0.143		−0.172		−0.064	
		*Sutterella*	**−0.406**	I	−0.247		**−0.612**	I	**−0.674**	**	0.045		0.107		−0.246		−0.099	
	*Enterobacteriaceae*		**−0.689**	*	**−0.761**	*	**−0.696**	*	**−0.775**	**	−0.045		−0.164		−0.266		0.123	
	*Pasteurellaceae*		**−0.553**	*	−0.313		**−0.745**	*	−0.313		−0.225		**0.437**	I	−0.222		−0.399	I
		*Haemophilus^c^*	**−0.475**	*	−0.264		**−0.735**	*	−0.330		−0.250		**0.441**		−0.169		**−0.466**	*
Actinobacteria	*Coriobacteriaceae*		0.304		0.209		0.248		−0.180		0.364	I	−0.101		−0.076		0.265	
		*Adlercreutzia*	**0.568**	*	**0.678**	*	**0.524**		−0.026		**0.403**	*	0.046		0.265		0.324	
		*Eggerthella*	−0.135		−0.353		0.013		−0.228		−0.043		−0.379		**−0.495**	I	0.144	
	*Actinomycetaceae*		−0.239		−0.191		**−0.420**		**−0.457**	I	0.180		0.236		0.007		−0.021	
		*Actinomyces*	−0.267		−0.124		−0.365		**−0.418**	I	0.116		0.148		−0.113		−0.082	
Verrucomicrobia	*Verrucomicrobiaceae*		**0.469**	I	0.358		**0.476**		0.129		0.170		−0.105		−0.015		0.177	
		*Akkermansia^d^*	**0.417**	I	0.394		**0.466**		0.105		0.127		−0.104		−0.003		0.090	
Synergistetes	*Synergistaceae*		**0.478**	I	**0.575**	I	0.292		0.169		0.097		0.289		0.132		0.004	
Fusobacteria	*Fusobacteriaceae*		−0.357		−0.206		**−0.615**	I	−0.196		−0.206		0.241		−0.161		−0.356	
		Fusobacterium	−0.363		−0.212		**−0.538**	I	−0.179		−0.228		0.191		−0.275		−0.380	

^a^*R. gnavus*; ^b^ *F. prausnitzii*; ^c^ *H. parainfluenzae*; ^d^ *A. muciniphila.*

Compared to microbiota footprint of healthy individuals (Table 3), both IBD and IBS had a marked increase in the abundance of Proteobacteria, most notably *Enterobacteriaceae*, as well as *Eubacterium*. Relative enrichment of *Dialister* was noted in CD and UC patients. At the same time, IBD specimens (CD in particular) display decreased abundance of several families belonging to the order *Clostridiales*, most notable being the depletion of *Christensenellaceae*. IBD affected individuals had several protective taxa depleted, particularly a significant depletion of *Anaerostipes* and *Ruminococcus*, a marked reduction of *A. muciniphila*, and a moderate decrease of *F. prausnitzii. Adlercreutzia* and *Lactobacillus* were also depleted in IBD. In CD patients, depletion of *Veillonellaceae* is noted, with significant reduction in the abundance of *Phascolarctobacterium* genus. *R. gnavus* was more abundant in all patients compared to healthy group, reaching statistical significance in CD. Although there is an increase in taxa belonging to the Bacteroidetes phylum across patient groups, the most prominent difference distinguishes IBS from healthy controls.

The capacity of fecal microbiota to discriminate among three disease groups is lower than between the disease and the healthy groups (Table 3). UC group had considerably higher abundance of *Turicibacteriaceae/Turicibacter* and *Haemophilus*. The significant decrease of *Veillonellaceae* differentiated between CD and UC, while *Erysipelotrichaceae, R. gnavus, Blautia* and *Coprococcus* were enriched in CD, especially when compared to IBS. In IBS specimens *Barnesiellaceae* were reduced compared to both IBD and healthy control.

### Comparison of gut mucosa and feces microbiota composition

**Figure 6.**
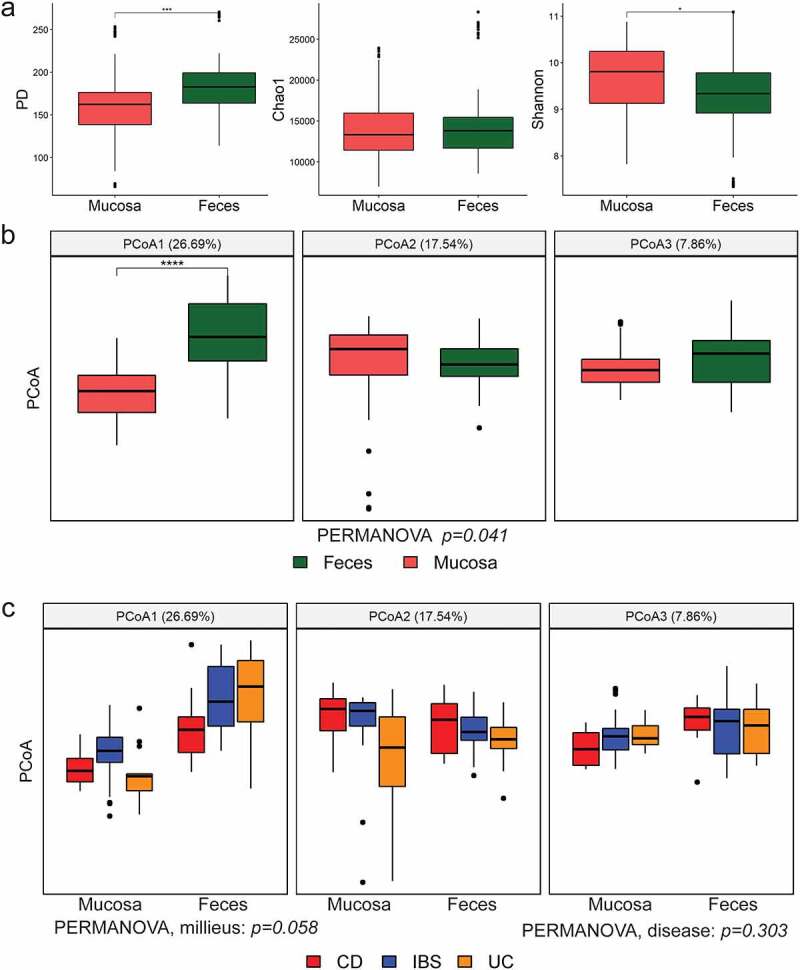
Microbial diversity indices per sample type. (a) Alpha diversity indices – PD, Chao1 and Shannon, with B.-H. corrected Wilcoxon test between categories. (b) and (c) beta diversity PCoA in weighted UniFrac per sample type (feces or mucosa) with Bonferroni-corrected t-test between categories, and separated by disease (CD, UC, IBS) for each specimen type, respectively. The number in brackets represents percentage of total variance explained by the given PCoA. *p < .05, **p < .01, ***p < .001, ****p < .0001.

Finally, profiles observed in the fecal microbiota were matched with profiles of intestinal mucosa of the same cohort. The highest contribution to the variance is the effect of the two different milieus, i.e. mucosa versus feces (PCoA1 in Figure 6b), with lesser contribution of diseases within each milieu. Interestingly, the impact of the disease on the variance is more distinct in mucosal samples (PCoA1 in Figure 6c).

When the contribution of individual taxa to the compositional changes between milieus was considered (Table 4), Actinobacteria and Fusobacteria were increased in mucosa, while Bacteroidetes were enriched in feces. A substantial number of families differentially abundant between milieus were detected, most notably *Bacillaceae* and *Propionibacteriaceae*, both highly specific for mucosal samples and virtually undetected in feces (with effect sizes >1). *Lachnospiraceae, Enterobacteriaceae, Erysipelotrichaceae, and Pasteurellaceae* were also more abundant in mucosa, while *Prevotellaceae, Bifidobacteriaceae, Rikenellaceae*, and *Clostridiaceae* were more abundant in feces. Despite marked differences in abundance between sample types, a set of core taxa (defined as present in >95% of sampled participants) in both mucosa and feces were identified, and accounted for >90% of abundance in each sample (Supplementary Table S4).

**Table 4.** Family abundance trends in patients’ feces (F) and mucosa (M) microbiota profiles presented as effect size (ES) and p-values in Kruskal-Wallis test, after BH correction for multiple testing; *** p < .001; ** p < .01; * p < .05

Gram-positive bacteria					Gram-negative bacteria				
Phylum	Family		ES	p	Phylum	Family		ES	p
Firmicutes	*Bacillaceae*	M > F	1.169	***	Bacteroidetes	*Prevotellaceae*	M < F	0.352	**
	*Peptostreptococcaceae*	M < F	0.934	***		*Rikenellaceae*	M < F	0.343	*
	*Planococcaceae*	M > F	0.695	***	Proteobacteria	*Pseudomonadaceae*	M > F	0.876	***
	*Gemellaceae*	M > F	0.663	***		*Moraxellaceae*	M > F	0.834	***
	*Carnobacteriaceae*	M > F	0.635	***		*Comamonadaceae*	M > F	0.735	***
	*(Tissierellaceae)*	M > F	0.397	*		*Sphingomonadaceae*	M > F	0.681	***
	*Lachnospiraceae*	M > F	0.381	*		*Neisseriaceae*	M > F	0.592	***
	*Turicibacteraceae*	M < F	0.375	*		*Pasteurellaceae*	M > F	0.420	***
	*Clostridiaceae*	M < F	0.328	*		*Rhizobiaceae*	M > F	0.500	**
	*Aerococcaceae*	M > F	0.388	*		*Phyllobacteriaceae*	M > F	0.482	*
	*Staphylococcaceae*	M > F	0.365	*		*Rhodobacteraceae*	M > F	0.465	**
	*Erysipelotrichaceae*	M > F	0.337	*		*Enterobacteriaceae*	M > F	0.459	**
Actinobacteria	*Propionibacteriaceae*	M > F	1.043	***		*Oxalobacteraceae*	M > F	0.444	**
	*Micrococcaceae*	M > F	0.891	***		*Bradyrhizobiaceae*	M > F	0.347	*
	*Corynebacteriaceae*	M > F	0.689	***		*Brucellaceae*	M > F	0.331	*
	*Actinomycetaceae*	M > F	0.647	***	Fusobacteria	*Fusobacteriaceae*	M > F	0.657	***
	*Bifidobacteriaceae*	M < F	0.345	*					

### Contribution of enterotype to microbiota composition

In feces, two major enterotypes were identified (*Bacteroides*- and *Prevotella*-enriched) with nine samples found outside of known enterotype space (no enterotype), (Supplementary Table S5). Clustering of effect sizes revealed a distinct pattern differentiating samples based on the enterotype (Figure 4, cluster 6 and Figure 5 cluster 1). Both enterotypes display similar PD and Chao1, while *Prevotella*-dominated specimens have lower Shannon diversity (Supplementary Figure S8a). The same trend in Shannon diversity was not observed in mucosa (Supplementary Figure S8b). The contribution of enterotype to beta diversity of fecal profiles was more pronounced than in mucosal ones (Supplementary Figure S8c), thus revealing greater robustness of mucosal samples to the enterotype.

## Discussion

In this report, we present a comprehensive insight into microbiota composition along the gut mucosa and in feces of newly diagnosed, treatment-naïve adult CD and UC patients in comparison with IBS controls. Our study investigated microbiota with respect to six anatomical sites, inflammation status of mucosa (inflamed or uninflamed) and patient diagnosis, as well as the relation of mucosal and fecal profiles. Fecal microbiota was further compared to those of healthy individuals, of whom mucosal samples were not obtained for ethical reasons. The demographics (age, sex, BMI) was generally balanced across the groups. Gender distribution in IBD was dependent on the disease subtype, with greater prevalence of female participants in CD group (1.4 to 1 ratio), and no significant gender differences in UC group, corresponding to the ratios seen in large population-based studies in the Europe and USA.^35,36^

One of the major findings of this study is the spatial homogeneity of microbiota along the colon based on the lack of community level differences or specific microbial structure that would characterize an anatomical site, as previously shown at 2 to 4 anatomical sites along the gut in IBD patients undergoing treatment.^24,26,30,31,37^ However, our study provides the most detailed coverage of the gut mucosa at six positions, and crucially timing is set at the disease onset.

Microbiota composition of inflamed and uninflamed sites was in high agreement, with no significant differences in alpha or beta diversity, as confirmed by high homogeneity and comparable distribution of detected families. A similar concordance between inflamed and uninflamed mucosa has been reported in pediatric^38^ and adult^26,28^ UC and CD patients with established disease.

This suggests that IBD inflammation onset and progression are not primarily associated with the present bacterial composition, but rather overall dysbiosis and its metabolic potential.

The principal coordinate of beta diversity for mucosal samples corresponded well with the sequencing depth (Supplementary Figure 4), meaning that other trends in the data were shadowed by this effect. We argue that pooling the samples of the same patient, as opposed to common practice of removing the samples based on the predefined cutoff of minimal sequence count (e.g. Forbes et al.^26^), is the favorable strategy to address this common issue, as the profiles of all positions within each patient are well correlated. Finally, by focusing on the unique profile of each donor upon pooling all the mucosal positions, and thus utilizing the entirety of available data we mitigated the effect on diversity of the lowest depth samples while keeping the specific profiles corresponding to disease of each participant in this study. Based on our findings and observed technical constraints of mucosal tissue sampling, we would encourage collection of a larger sample either from a single or multiple colon segments, in order to increase the read depth and improve the microbiota coverage and consistency. Eventually, creating a single mucosal microbiota profile for each patient facilitates comparison with the fecal profile of the individual.

We detected a distinctive mucosal microbiota footprint of treatment-naïve IBD patients when compared to IBS, with alpha diversity severely reduced in IBD. Beta diversity further distinguished between IBD (UC in particular) and IBS. The microbiota profiles on family and genus levels discriminate IBD from IBS and abundance of several taxa can discern between CD and UC. In IBD patients, significant enrichment in *Dialister, Propionibacterium* and *Haemophilus* was observed, while the most distinctive feature between CD and UC was a significantly higher abundance of *R. gnavus* and *Blautia* in CD. Similar trends were reported in IBD patients undergoing diverse therapeutic regimens indicating that these microorganisms could play an important role in CD pathogenesis.^24,39^

Fecal footprints differentiated well amongst healthy individuals and those with gastrointestinal disorders even at the disease onset, supporting the relevance of microbiota changes in the early pathogenesis of IBD as well as of IBS, but have lower power to discriminate among these diseases. Moreover, the major contributor to the variance in fecal microbiota diversity was the enterotype, while its effect was less evident in mucosal profiles, exposing more clearly the disease-specific patterns.

When comparing fecal abundances between all four groups, the most prominent shifts were found in Firmicutes phylum. *Christenellaceae, Ruminococacceae, Anaerostipes* and *Adlercreutzia* were depleted in CD and UC, all taxa associated with microbiome of healthy individuals.^28,40–42^ Compared to healthy individuals increased abundance of *Lactobacillaceae* was found in patient groups, consistent with prior studies.^30,43,44^ Significant increase of *Turicibacter* found in UC patients, to our knowledge, has not been reported so far. Depletion of *Turicibacter* was, however, found in a limited cohort of new-onset non-Western pediatric CD patients.^45^ While observed reduction of *Phascolarctobacterium* in IBD is in keeping with studies linking its decrease to colonic inflammation,^46^ overabundance of *Eubacterium* contrasts the reported reduction in pediatric and adult IBD patients.^27,47,48^

Depletion of SCFA producing bacteria is associated with aberrant immune responses and impaired intestinal barrier integrity.^22,49^ Decrease in butyrate-producers (e.g. Clostridium cluster XIVa genera *Blautia, Coprococcus, Dorea* and *Roseburia*, as well as *F. prausnitzii* and *Anaerostipes*) with concomitant expansion of Proteobacteria is often reported in IBD and IBS patients.^17,18,44,47,50^ In feces of IBD patients from our cohort, we observed a significant reduction of *Anaerostipes* genus, but the effect of *F. prausnitzii* reduction was small, although the reduction of *F. prausnitzii, Roseburia* and *Ruminococcus* was previously reported in feces of treatment-naïve adult CD patients.^51^ *F. prausnitzii* was depleted in mucosa of IBD patients with active disease,^27,30,52^ but this trend was not replicated in our study, as biopsies of healthy controls were not available for comparison. Still, lower abundance of other butyrate producers was found in biopsies of UC patients. Reduction of *A. muciniphila*, a constituent of healthy microbiota important for the maintenance of mucus layer,^53,54^ was observed in feces of IBD and mucosa of CD patients. Significant decrease of *Adlercreutzia* found in feces of CD and UC supports recent observations in UC patients.^40,42^ *Adlerkreutzia* genus metabolizes isoflavones, phenolic compounds with antimicrobial and anti-inflammatory properties,^55^ so its reduction may promote inflammation. In addition, we also observed a previously unreported decrease of *Adlerkreutzia* in mucosa of CD patients.

Alterations of lipid profiles in IBD involve other microbiota-derived fatty acids as well.^21,56^ One of the hallmarks of UC in our study was the expansion of *H. parainfluenzae*, which has been associated with increased level of acylcarnitine in IBD.^57^ Expansion of *H. parainfluenzae* was found in pediatric UC patients,^32,38,58^ and its reduction in colonic biopsies of adult UC patients in response to novel anti-TNF neutralizing antibody has been reported,^59^ suggesting a potential role of *H. parainfluenzae* in IBD pathogenesis. The enrichment of *Haemophilus* in biopsies of CD patients was reported in the pediatric treatment-naïve cohorts,^27,60^ but we did not observe a similar trend.

The expansion of potentially harmful pathobiont *R. gnavus*, which contributes to gut inflammation through production of proinflammatory polysaccharide and degradation of mucosal barrier,^61^ was detected in both feces and mucosa of CD group, consistent with previous reports.^24,39^ *Dialister* has recently emerged as a genus of potential interest in IBD, but conclusive evidence on its role is still lacking. We found it enriched in mucosa and feces of IBD patients, in agreement with recent study comparing rectal biopsies of IBD patients to healthy controls.^23^ In contrast, reduction of *Dialister* has been reported in feces of newly diagnosed^62^ and a small cohort of established pediatric CD patients,^63^ where lower abundance of *Dialister* was associated with increased calprotectin levels. We also detected an increase of *Propionibacterium acnes*, a known member of skin microbiota, in mucosa of UC patients. Increased *P. acnes* was found in biopsies of newly diagnosed pediatric CD patients,^60^ and its enrichment in gastric microbiota was associated with increased risk of gastric cancer.^64^

Present report is, to our knowledge, the first comparison of fecal and mucosal microbiota in adult CD, UC and IBS patients at the onset of disease. The rigorous inclusion and exclusion criteria allowed us to determine microbiota composition prior to any influence of antimicrobial or anti-inflammatory therapy on the bacterial content. The two are known to drive and silence the dysbiotic state in IBD,^27,32,65,66^ so we found it crucial to examine treatment-naïve patients in order to explore the role of microbiota in IBD onset. By employing state-of-the art statistical approaches highly amenable to compositional nature of the microbiota data,^67^ we present only the most robust and biologically relevant features of the data that do not only conform to previously detected trends (both by 16S and shotgun metagenomics methodologies),^12^ but also highlight some of the yet underexplored genera.

We show that the microbiota composition of gut mucosa is spatially homogeneous from terminal ileum to rectum of each patient, cannot be differentiated based on the local inflammation status of the mucosa, and yet provides disease-specific footprints. Both milieu footprints could be reduced to a core set of taxa, even on the small cohort presented here. Microbiota core to large extent corresponded well in both feces and mucosa and consisted of taxa usually found in European population.^10^ The results obtained justify further investigation of the taxa discriminating UC, CD and IBS groups, such as *H. parainfluenzae, R. gnavus, Turicibacteriaceae, Dialister* and *Adlercreutzia*, as potential biomarkers of the disease.

The fecal microbiota in treatment-naïve adult IBD and IBS patients can be clearly distinguished from the healthy individuals. While fecal profiles did show discernible trends in abundances in each of the groups (healthy, IBS, CD, UC), these trends were only partially consistent and therefore not directly transferable to the mucosal microbiota profiles of the same cohort. Unsurprisingly and in concurrence with suggested higher discriminatory power of mucosa-associated microbiota for classifying the disease,^25,27^ our results provided superior resolution between patient groups on mucosal samples, than on fecal ones.

We thus suggest that the mucosal profiles, as these are more stable and less influenced by environmental factors like food consumed and other xenobiotics, may provide more accurate insight into the role of gut microbiota in IBD pathobiology, however one cannot neglect the fact that this kind of sampling involves an invasive endoscopic procedure. Finally, we confirm many of the previously detected trends reflecting IBD gut microbiota dysbiosis, but in this case on the newly diagnosed treatment-naïve adults, indicating that the changes in mucosal and fecal microbiota are set and consistent along the gut mucosa, and detectable at the disease onset and likely have a discerning role in IBD pathophysiology.

## Patients, materials, and methods

The research was conducted at the Center for Translational and Clinical Research, University of Zagreb School of Medicine (UZSM), and at the Department of Gastroenterology, University Hospital Center Zagreb (UHCZ) and patients were recruited from 2014–2018.

### Study population

Adult participants that presented with gastrointestinal symptoms associated with IBD (such as diarrhea, abdominal pain and blood or mucus in stool for >2 weeks), with no exposure to any IBD related medical therapies or antibiotics were included in the study. All participants signed an informed consent form prior to sample collection and all procedures were carried out in accordance with the approved study protocol (Ethics Committee of the UZSM, case number: 380–59-10106-14-55/149 and UHCZ, case number: 02/21/JG). Participants’ personal pseudoanonymized data were stored in electronic form, and researchers fully complied with prescribed procedures for personal data protection. After collection of samples, participants’ diagnoses were subsequently determined following established diagnostic procedures and they received treatment appropriate for their condition.

Criteria for inclusion in the study were: minimum age of eighteen; no treatment for inflammatory bowel disease (5-aminosalicylic acid, corticosteroids, TNF-α antibodies, azathioprine, 6-mercaptopurine, etc.) in the medical history; no antibiotic treatment for at least three months prior to recruitment. Exclusion criteria were: confirmed pregnancy at the recruitment time point or planning to become pregnant; coronary heart disease, diabetes, chronic obstructive pulmonary disease, chronic renal failure, malignant diseases, autoimmune diseases or any severe chronic disease, addiction, severe psychiatric illness in the medical record (past or present); subjects for whom the researcher estimates that for any reason they will not be able to adequately cooperate in the study. Patients fitting these criteria were then diagnosed at the UHCZ according to clinical, endoscopic, histological and radiological criteria and were divided into three groups: the patients with either CD or UC diagnosis comprised the IBD study group, while the subjects newly diagnosed with the IBS defined the control group. A total of 39 patients subsequently diagnosed as IBS were recruited, out of which 26 patients age-matched to the CD and UC group were included in this study, eliminating 13 IBS patients. No information on IBS subtypes, i.e. IBS-C vs IBS-D was recorded during recruitment. All other diagnoses were excluded from further consideration. To characterize the phenotypes of CD and UC Montreal classification was used, while endoscopic activity at the diagnosis was assessed according to SES-CD score for CD and Mayo score for UC.^34^ The physician performing endoscopy determined inflammation status of the specimens. IBS patients were diagnosed according to Rome III criteria.

We also recruited 12 healthy donors with no history of gastrointestinal symptoms associated with IBD, additional to all exclusion criteria pertaining to the study, and obtained their fecal samples. None of the participants reported the use of probiotics or prebiotics. Basic demographic data of study participants are presented in Table 1.

### Sample collection

Colonic mucosa biopsy samples from enrolled participants were collected in the course of diagnostic endoscopic procedure according to the standard hospital protocol, on bowels cleansed with MoviPrep® for previous 24 h. Samples were collected at six distinct anatomical sites along the gut (Figure 2): terminal ileum (TI), colon ascendens (CA), colon transversum (CT), colon descendens (CD), colon sigmoideum (CS), and rectum (R), and stored at −80°C.

Feces was collected and stabilized in the home setting prior to colon cleansing and hospital visit for diagnostic endoscopic procedure, using OMNIgene.GUT fecal collection kit (DNA Genotek, Cat. OMR200), according to the manufacturer’s instructions and was further processed at the earliest opportunity (within 7 days after collection).

### DNA extraction and sequencing

DNA from mucosal specimens was extracted using MasterPure DNA purification kit (Epicenter, Cat. MC85200). Samples were homogenized in a Minilys homogenizer (Bertin technologies, Montigny-le-Bretonneux, France) using Soil grinding kit SK38 (Bertin Pharma, Cat. D34016) and proteinase K (MP Biomedicals, Cat. 193504), followed by the manufacturer’s recommended protocol for DNA extraction.

Fecal DNA was extracted using MP Biomedicals Fast DNA spin kit for feces (MP Biomedicals, Cat. 116570200), following manufacturer’s instructions. Samples were homogenized in kit-supplied tubes prefilled with ceramic and silica particles, using Minilys homogenizer.

Extracted DNA was stored in TE buffer at −20°C. The DNA quantity and quality were determined based on the absorbance (Nanodrop 2000) and fluorescence (Qubit 3.0, both Thermo Fisher Scientific, Germany) measurements. DNA integrity was confirmed by agarose gel electrophoresis. Median DNA yield was 232 ng/µL (mean 308 ng/µL, interquartile range 162–376 ng/µL) for mucosal, and 70 ng/µL (mean 89 ng/µL, interquartile range 43–100 ng/µL) for feces samples.

DNA sequencing was performed as previously described.^68^ Briefly, Nextera XT DNA Sample Preparation Kit (Illumina, Cat. FC-131-1096) was used for construction of 16S libraries. PCR amplification of hypervariable regions V3-V4 of 16S rRNA gene resulted in a single amplicon with mean length of 464 bp.^69^ Paired-end sequencing was performed on MiSeq platform (Illumina CA, USA) with MiSeq Reagent Kit v3 (Illumina, Cat. MS-102-3003), according to the manufacturer’s instructions.

### Microbiota quantification and characterization

Raw sequencing files were processed using QIIME pipeline.^70^ Fastq files containing paired-end reads were merged, allowing overlap between mates (‘–allow-outies’ option), using FLASh,^71^ then trimmed, filtered by quality and chimeric sequences were removed as described in the default QIIME pipeline. Operational Taxonomic Units (OTUs) were assigned using usearch^72^ and PyNast alignment^73^ against the GreenGenes database (version 13_8).^74^ Cumulative OTU counts for each taxonomy level from phylum to genus were extracted into separate tables. The median number of reads in the final subsampled dataset used for all the analyses presented hereafter was 7,916 (mean 11,867, inter-quantile range 3,469–14,734) per mucosal specimen and 59,996 (mean 76,389, inter-quantile range 39,536–94,560) per feces specimen.

The compositional diversity within each sample was ascertained on rarefied sequences using a phylogeny-sensitive alpha diversity index Faith’s D (i.e. its alias termed PD), that accounts for the taxonomic distance, followed by the two phylogeny-agnostic indices, namely, Chao1 (species richness) and Shannon’s diversity (accounts for both abundance and evenness), as implemented in the QIIME pipeline. Significance of differences in mean alpha diversity were reported as Benjamini-Hochberg (B.-H.) corrected Wilcoxon test p-values. All alpha diversity values were calculated on samples rarefied to 2500 reads for mucosa samples before merging, and 10000 for merged mucosa and fecal samples. The compositional diversity between samples measured as beta diversity, was reported using quantitative, divergence-based weighted UniFrac distance measure^75^ and presented as principle coordinates (PCoAs), as implemented in the QIIME pipeline. The correspondence of measured attributes of the data (i.e. diagnosis, gut position, etc.) to the top PCoAs is reported as the Bonferroni-corrected p-values for the t-test between attribute classes. Homogeneity of variances between groups was tested with multivariate implementation of Levene’s test, as implemented in betadisper function in vegan R package in R. The permutational multivariate analysis of variance (PERMANOVA) was performed, based on weighted UniFrac distances using Adonis test with 999 permutations, as implemented in vegan R package.

The compositional aspect of the data^76^ was preserved and the statistical analyses were performed based on the appropriate methodology.^77–79^ To identify differentially abundant taxa, we applied ALDEx2 (ANOVA-like differential expression analysis) R package. Briefly, the approach is based on the centered-log-ratio (clr) sequencing count data transformation to ensure compositionally coherent inference using iqlr-based denominator, and on 128 Monte Carlo Dirichlet instances to control for type-I error due to the underestimated variance of low abundance taxa.

Each fecal sample was classified into one of the three predicted enterotypes, based on the classification proposed by Arumugam et al,^15^ by uploading genus level counts into the online tool at https://enterotypes.org/. There were no samples detected with *Ruminoccocus*-enriched enterotype, and nine samples not falling within the enterotype space (i.e. “within_ET_space” label set to FALSE) are excluded, when enterotype-based results are presented.

ALDEx2 was also used to calculate both the effect size of the difference between groups for each taxon, and the significance of the contribution of each taxon using Kruskal-Wallis (KW) test. Pairwise comparisons were made for the different combinations of disease groups, inflammation status and position along the gut (proximal and distal). Uncorrected p-values for the KW test are reported and symbolized by ** p < .01; * p < .05; I p < .1 (for indicative, approaching statistical significance).

All metrics are reported on the family level, unless otherwise indicated. Even though sequencing of 16S rRNA gene amplicons does not generally provide accurate identification of bacteria on the species level,^80,81^ owing to sufficient sequencing depths of some taxa, we were able to discriminate *Ruminococcus gnavus, Haemophilus parainfluenzae, Veillonella dispar, Akkermansia muciniphila*, and *Fecalibacterium prausnitzii*.

Heatmap was based on effect sizes and clusters were determined using k-means clustering, as implemented in R programming language^82^ with 3–9 clusters considered, and the best number of clusters was chosen upon manual inspection. In the abundance annotation column, families were sorted by overall abundances in the entire dataset in four logarithmic bins, for more details, see Supplementary Table 1.

Principal component analysis (PCA) is shown on transformed counts using the ‘prcomp’ function in R. The PCA biplots show only a subset of loadings for clarity: from the set of significant families, only the top ten most abundant (based on their total abundance in mucosal microbiota) are shown.

## Supplementary Material

Supplemental Material

## Data Availability

The sequencing data that support the findings of this study are openly available in Figshare repository at https://doi.org/10.6084/m9.figshare.17012411 and https://doi.org/10.6084/m9.figshare.17012558.
