# Supplementary material for: Gut microbiota in mucosa and feces of newly diagnosed, treatment-naïve adult inflammatory bowel disease and irritable bowel syndrome patients
Source: Gut Microbes. 2022 Jun 13;14(1):2083419. doi: 10.1080/19490976.2022.2083419 (PMC9196785; doi:10.1080/19490976.2022.2083419)
Supplement: Supplemental Material [file KGMI_A_2083419_SM1148.zip › Paljetak_GM1_Supplementary_material_rev2.docx]

**Supplementary information for:**

**Gut Microbiota in Mucosa and Feces of Newly Diagnosed, Treatment-naïve Adult Inflammatory Bowel Disease and Irritable Bowel Syndrome Patients**

Hana Čipčić Paljetak, Anja Barešić, Marina Panek, Mihaela Perić, Mario Matijašić, Ivana Lojkić, Ana Barišić, Darija Vranešić Bender, Dina Ljubas Kelečić, Marko Brinar, Mirjana Kalauz, Marija Miličević, Dora Grgić, Nikša Turk, Irena Karas, Silvija Čuković-Čavka, Željko Krznarić, Donatella Verbanac

Supplementary figures S1 to S8

Supplementary tables S1, S4 and S5

Supplementary tables S2 and S3 are in a separate Excel file


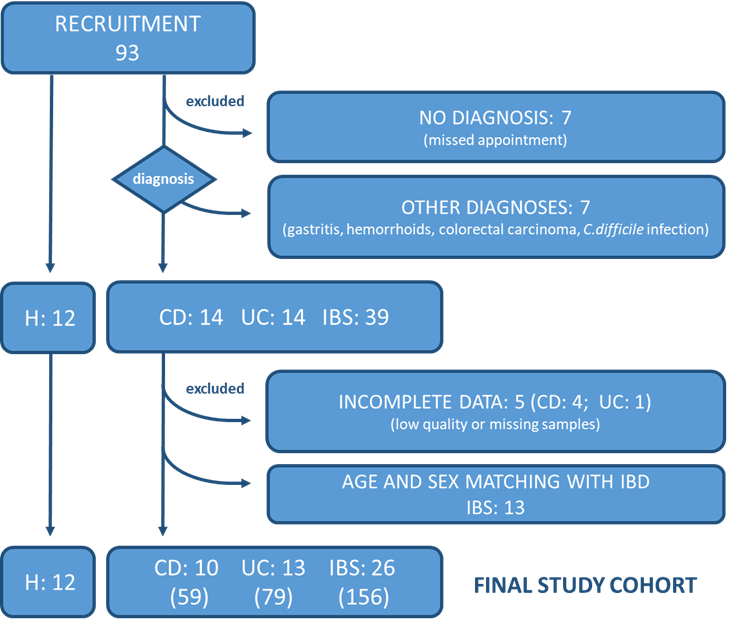


Supplementary figure S1. Scheme of the recruitment procedure. Patients were excluded from the downstream analyses if not diagnosed, diagnosed with conditions other than IBS or IBD, due to IBS matching and for other technical reasons. The numbers in brackets for the final study cohort refer to the total number of mucosal samples collected for the included patients in that group. A single fecal sample was collected from all participants.


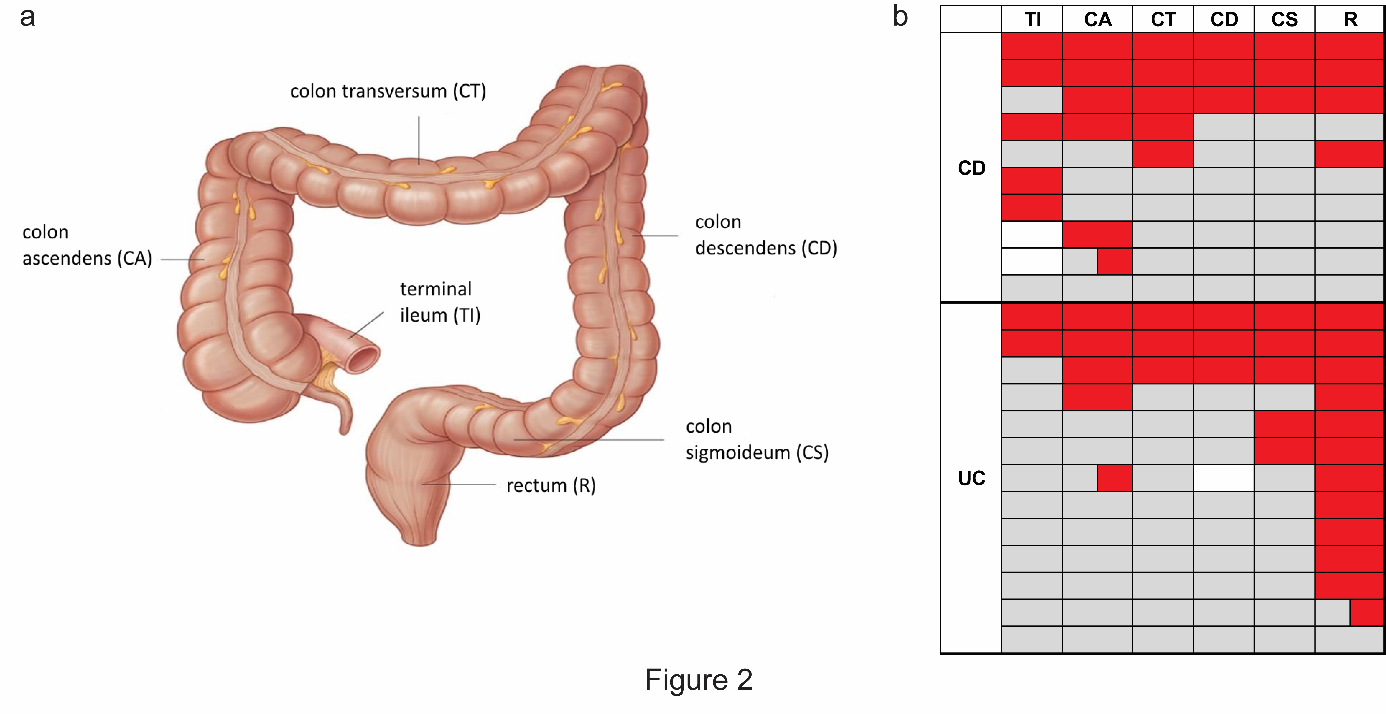


Supplementary figure S2. Study design. (a) Sampling sites along the gut. (b) Sampled gut mucosa positions in IBD patients, with inflammation status for anatomical sites along the gut. Inflamed positions are shown in red, uninflamed in grey, not sampled in white, and split grey/red shows positions where both inflamed and uninflamed samples were taken. For 26 IBS individuals, all six positions were sampled with no inflamed sites along the gut.


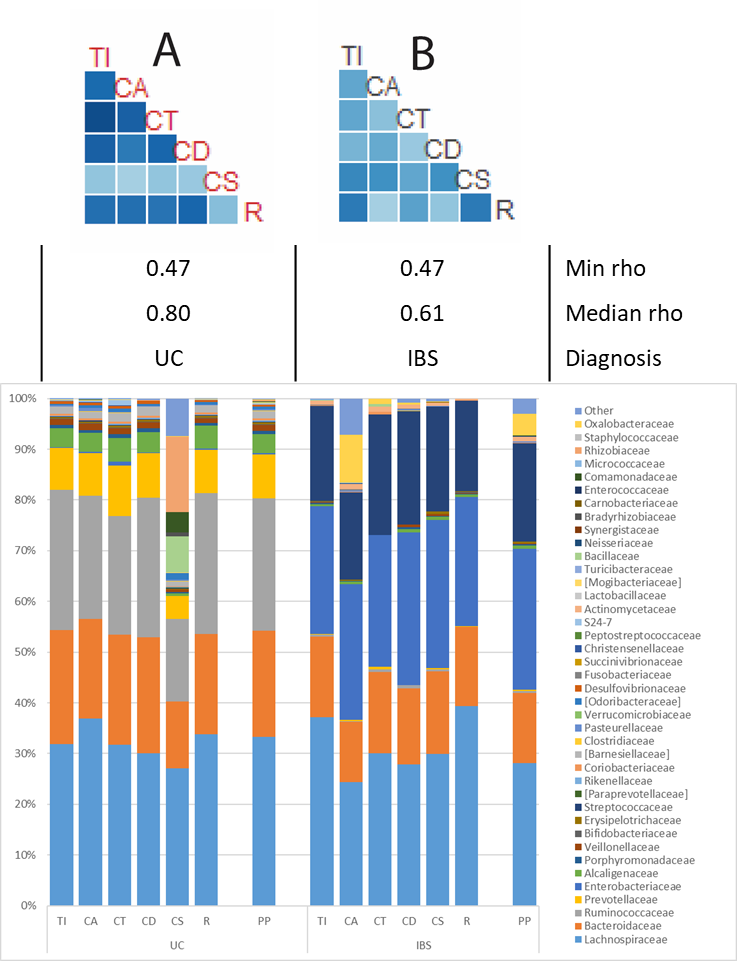


Supplementary figure S3. Relative abundance profiles for two examples of patients with within-patient per-position microbiota at the lower of the spectrum of Spearman’s correlation rho. Terminal ileum (TI), ascending colon (CA), transverse colon (CT), descending colon (CD), sigmoid colon (CS), rectum (R), patient profile after merging (PP).


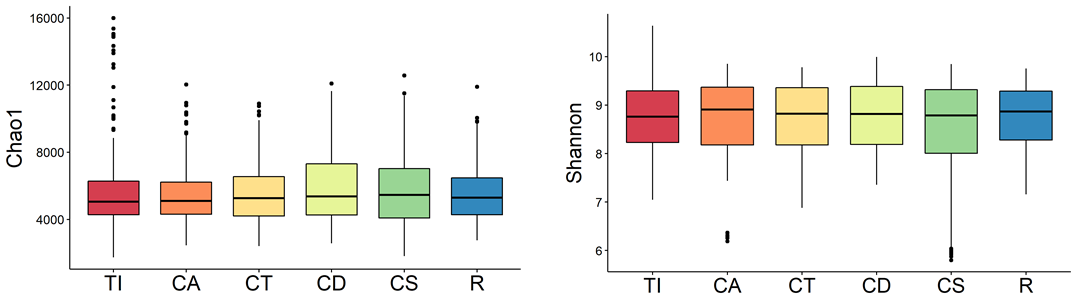


Supplementary figure S4. Microbial diversity of intestinal mucosa at six positions along the gut. B.-H. corrected Wilcoxon test between categories showed no significance. Chao1 estimates the species richness, while Shannon diversity index accounts for both species richness and evenness.


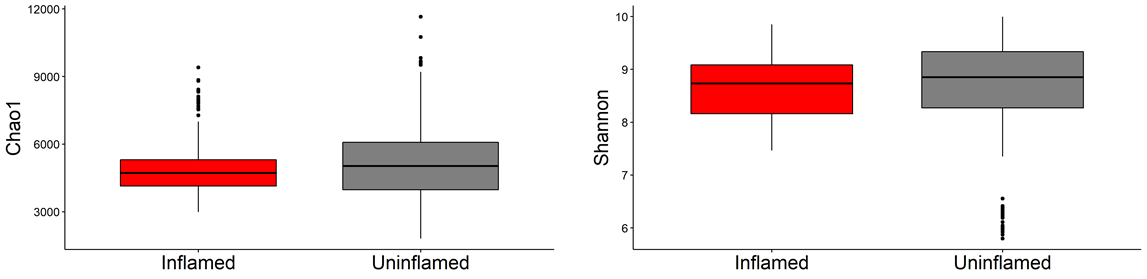


Supplementary figure S5. Microbial diversity of intestinal mucosa at inflamed and uninflamed positions along the gut. B.-H. corrected Wilcoxon test between categories showed no significance. Chao1 estimates the species richness, while Shannon diversity index accounts for both species richness and evenness.


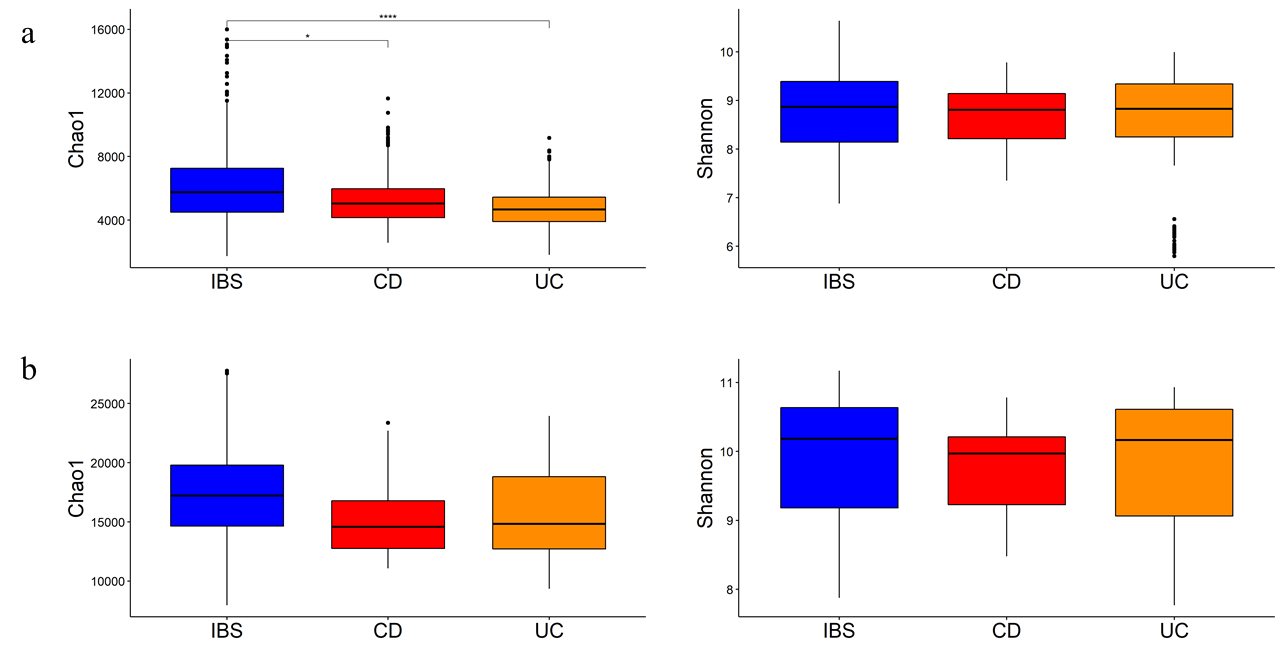


Supplementary figure S6. Microbial diversity of intestinal mucosa split by diagnosis at six positions along the gut (a), and after merging positions into patient-specific profiles (b), with B.-H. corrected Wilcoxon test between categories. Chao1 estimates the species richness, while Shannon diversity index accounts for both species richness and evenness. CD – Crohn’s disease, UC – ulcerative colitis, IBS – irritable bowel syndrome. *p<0.05, **p<0.01, ***p<0.001, ****p<0.0001.


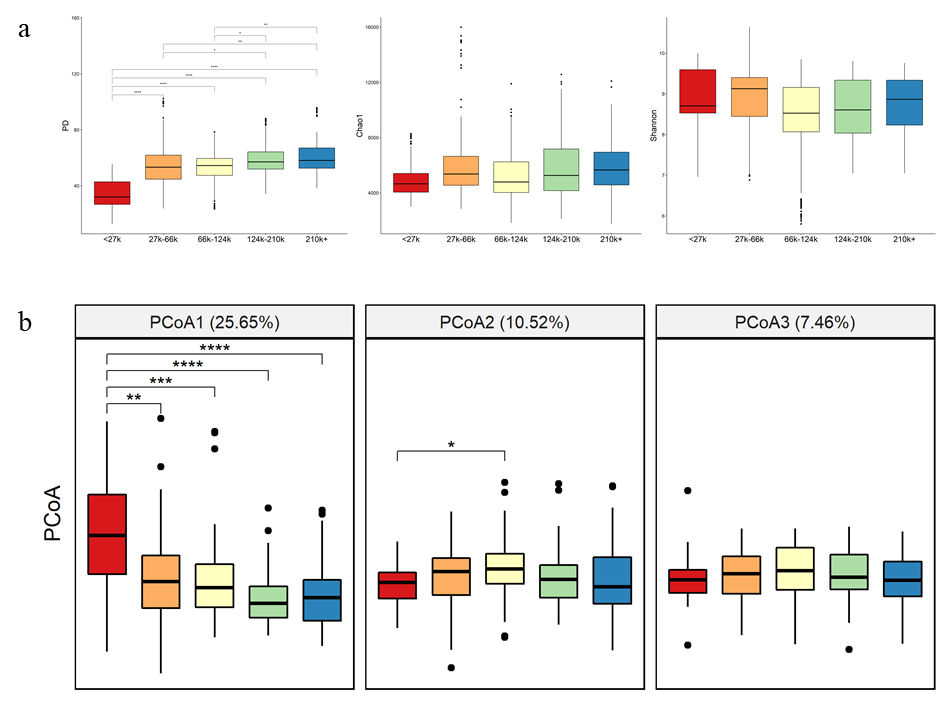


Supplementary figure S7. Alpha diversity indices for samples binned according to sequencing depth. (a) PD, Chao1 and Shannon alpha diversity indices, with B.-H. corrected Wilcoxon test between categories. (b) shows the top three PCoAs of beta diversity (percentage of the variation explained in brackets), colored by sequencing depth bins chosen to contain the same number of samples, with Bonferroni-corrected t-test between categories. *p<0.05, **p<0.01, ***p<0.001, ****p<0.0001.


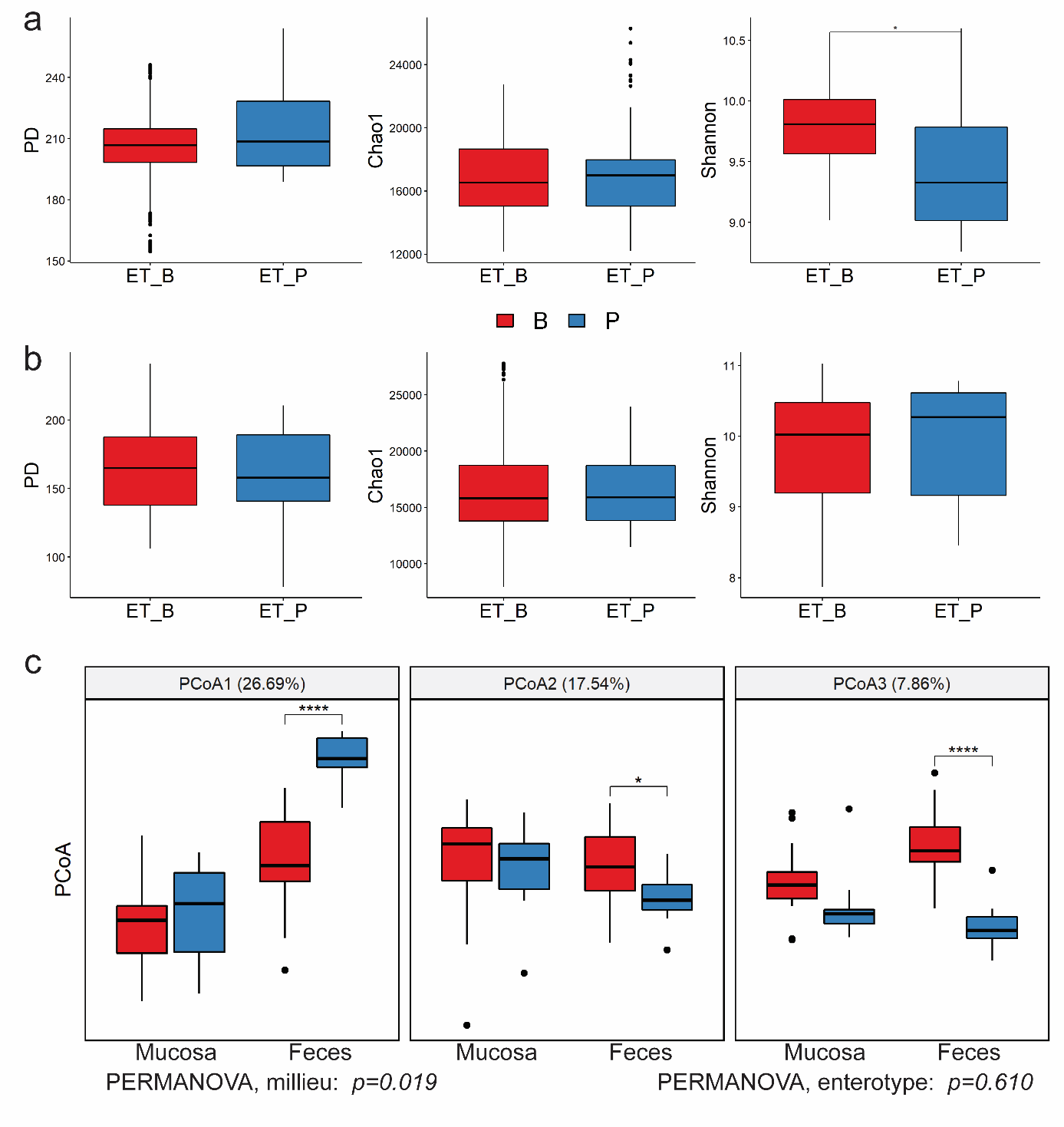


Supplementary figure S8. Enterotype-based analysis of microbiota diversity in feces and mucosa. B – *Bacteroides*-enriched enterotype; P – *Prevotella*-enriched enterotype. (a) alpha diversity indices in feces specimens, PD, Chao1 and Shannon, with B.-H. corrected Wilcoxon test between categories. (b) alpha diversity indices in feces specimens. (c) beta diversity PCoA in weighted UniFrac per enterotype in microbiota profiles of mucosa and feces, with Bonferroni-corrected t-test between categories. The number in brackets represents the percentage of total variance explained by the given PCoA. *p<0.05, **p<0.01, ***p<0.001, ****p<0.0001.

Supplementary table S1. Distribution of taxa in abundance bins for heatmap, according to the counts in the entire mucosal and fecal datasets.

| Abundance bin | Number of counts in a dataset |
| --- | --- |
| 1 | <100 |
| 2 | 100-10.000 |
| 3 | 10.000-100.000 |
| 4 | >100.000 |

Supplementary table S4. Bacterial taxa representing core microbiota. Constituents of core microbiota in each sample type, i.e. feces (F) and gut mucosa (M) are highlighted in grey.

| Phylum | order | family | genus | F | M |
| --- | --- | --- | --- | --- | --- |
| Actinobacteria | Actinomycetales | *Actinomycetaceae* |  |  |  |
|  | Bifidobacteriales | *Bifidobacteriaceae* | *Bifidobacterium* |  |  |
|  | Coriobacteriales | *Coriobacteriaceae* | *Collinsella* |  |  |
| Bacteroidetes | Bacteroidales | *[Odoribacteraceae]* | *Odoribacter* |  |  |
|  |  | *Bacteroidaceae* | *Bacteroides* |  |  |
|  |  | *Porphyromonadaceae* | *Parabacteroides* |  |  |
|  |  | *Prevotellaceae* | *Prevotella* |  |  |
|  |  | *Rikenellaceae* |  |  |  |
| Firmicutes | Lactobacillales | *Streptococcaceae* | *Streptococcus* |  |  |
|  | Clostridiales | *Clostridiaceae* | *Clostridium* |  |  |
|  |  |  | *SMB53* |  |  |
|  |  | *Lachnospiraceae* | *[Ruminococcus]* |  |  |
|  |  |  | *Blautia* |  |  |
|  |  |  | *Coprococcus* |  |  |
|  |  |  | *Dorea* |  |  |
|  |  |  | *Lachnospira* |  |  |
|  |  |  | *Roseburia* |  |  |
|  |  | *Peptostreptococcaceae* |  |  |  |
|  |  | *Ruminococcaceae* | *Faecalibacterium* |  |  |
|  |  |  | *Oscillospira* |  |  |
|  |  |  | *Ruminococcus* |  |  |
|  |  | *Veillonellaceae* |  |  |  |
|  | Erysipelotrichales | *Erysipelotrichaceae* |  |  |  |
| Proteobacteria | Burkholderiales | *Alcaligenaceae* | *Sutterella* |  |  |
|  | Desulfovibrionales | *Desulfovibrionaceae* | *Bilophila* |  |  |
|  | Enterobacteriales | *Enterobacteriaceae* |  |  |  |
|  | Pasteurellales | *Pasteurellaceae* | *Haemophilus* |  |  |
| Fusobacteria | Fusobacteriales | *Fusobacteriaceae* | *Fusobacterium* |  |  |
|  |  |  |  |  |  |
| median abundance in all faeces | | 98.20% | 94.30% |  |  |
| median abundance in all mucosa | | 96.14% | 92.50% |  |  |

Supplementary table S5. Enterotype distribution in feces stratified by disease.

|  | CD | UC | IBS | H | all |
| --- | --- | --- | --- | --- | --- |
| ET_B (%) | 5 (50) | 7 (54) | 16 (62) | 8 (67) | 36 (59) |
| ET_P (%) | 2 (20) | 2 (15) | 7 (27) | 4 (33) | 15 (25) |
| No_ET (%) | 3 (30) | 3(23) | 3 (12) | 0 (0) | 9 (15) |
| B/P ratio | 2.5 | 3.5 | 2.3 | 2.0 | 2.4 |
